# BORA regulates cell proliferation and migration in bladder cancer

**DOI:** 10.1186/s12935-020-01392-8

**Published:** 2020-07-06

**Authors:** Songtao Cheng, Tianchen Peng, Xiaolu Zhu, Fenfang Zhou, Gang Wang, Lingao Ju, Yu Xiao, Xuefeng Liu, Xinghuan Wang

**Affiliations:** 1 Department of Urology, Zhongnan Hospital of Wuhan University, Wuhan, China; 2 Department of Pathology, Lombardi Comprehensive Cancer Center, Georgetown University Medical School, Washington, DC USA; 3 Cancer Precision Diagnosis and Treatment and Translational Medicine Hubei Engineering Research Center, Wuhan, China; 4 Department of Ophthalmology, Zhongnan Hospital of Wuhan University, Wuhan, China; 5 Department of Biological Repositories, Zhongnan Hospital of Wuhan University, Wuhan, China; 6 Human Genetics Resource Preservation Center of Wuhan University, Wuhan, China; 7 Human Genetics Resource Preservation Center of Hubei Province, Wuhan, China

**Keywords:** Bladder cancer, BORA, Proliferation, Cell cycle, Epithelial-mesenchymal transition

## Abstract

**Background:**

Bladder cancer is having a gradually increasing incidence in China. Except for the traditional chemotherapy drugs, there are no emerging new drugs for almost 30 years in bladder cancer. New potential therapeutic targets and biomarkers are urgently needed.

**Methods:**

BORA is the activator of kinase Aurora A and plays an important role in cell cycle progression. To investigate the function of BORA in BCa, we established *BORA* knockdown and overexpression cell models for in vitro studies, xenograft and pulmonary metastasis mouse models for in vivo studies.

**Results:**

Our results indicated that BORA was upregulated in human bladder cancer (BCa) compared to the normal bladder and paracancerous tissues at transcriptional and translational levels. We found that BORA was positively related to BCa cell proliferation. Furthermore, *BORA* knockdown induced cell cycle arrest in G2/M phase while *BORA* overexpression decreased the proportion of cells in G2/M, associated with PLK1–CDC25C–CDK1 alteration. Interestingly, we observed that knockdown of *BORA* inhibited BCa cell migration and invasion, accompanied with alterations of epithelial–mesenchymal transition (EMT) pathway related proteins. In vivo studies confirmed the inhibition effect of *BORA* knockdown on BCa cell growth and migration.

**Conclusions:**

Our study indicates that BORA regulates BCa cell cycle and growth, meanwhile influences cell motility by EMT, and could be a novel biomarker and potential therapeutic target in BCa.

## Background

As the ninth most common cancer worldwide [1], bladder cancer (BCa) is having a gradually increasing incidence in China [2]. Most of the newly diagnosed cases are non-muscle invasive BCa. Even with transurethral resection of tumor, BCa still has a very high recurrence rate [3]. Chemotherapy based on cisplatin has improved the outcome modestly. For cisplatin-ineligible patients, T-cell checkpoint inhibitors have presented some benefits to those having high PD-L1 expression in some trials [4–6]. Except for the traditional chemotherapy drugs, there are no emerging new drugs for almost 30 years in BCa [7]. Therefore, to enhance the targeted and personalized therapy, molecular analysis to find more new specific markers and therapeutic targets is of great urgent.

*BORA* encoded protein activates kinase Aurora A, and is very important in spindle assembly, centrosome maturation and the process of mitosis. BORA was identified as a cell cycle co-factor protein of Aurora A in the first place [8]. Binding with pole-like kinase 1 (PLK1), BORA forms a PLK1/BORA complex and recruits Aurora A to the T-loop of PLK1 T210 phosphorylation site to activate PLK1, thus promote mitotic entry [9]. PLK1 and Aurora A are critical regulators of cell cycle, which has a fundamental role in cell proliferation, and related to the checkpoint recovery when DNA damage appears in cells where it leads to DNA repair or progress to apoptosis [10, 11]. A variety of cell cycle related regulators have been explored as therapeutic targets and biomarkers [12]. PLK1 and Aurora A inhibitors have been extensively explored over the last few years and some of them showed prospective clinical benefits [13–16]. Moreover, compounds affecting the interaction of BORA and PLK1 may also have a good therapeutic potential [17]. Zhang et al. revealed that BORA was overexpressed in lung, breast, and gastric adenocarcinomas, and was an independent biomarker associated with poor prognosis [18]. Furthermore, recent studies reported that BORA was significantly related to radiosensitivity by influencing DNA repair and MDC1 [19]. Therefore, the genome stability and cell cycle regulated by Aurora A/BORA/PLK1 axis have a great important role in tumorigenesis and progress [20]. The roles of Aurora A and PLK1 have been extensively explored in a variety of cancers. However, the expression of BORA and its effects on tumor biology are rarely reported especially in BCa.

Our group have screened a lot of differentially expressed genes through bioinformatics analysis of microarray data from BCa and normal bladder tissues [21, 22], and have verified several potential therapeutic targets and biomarkers associated with tumor progress and prognosis [23–26]. In the present study, we have verified that *BORA* was highly expressed in BCa compared to the normal bladder and paired paracancerous tissues, which was consistent with our microarray results. Further analysis indicated that BORA was positively associated with BCa cell proliferation. Knockdown of *BORA* induced cell cycle arrest in G2/M phase. Interestingly, we first found that reduced *BORA* repressed BCa cell mobility. Mouse model verified our in vitro results.

## Methods

### Ethical statement of human tissues

Bladder tissues were collected from the surgery of patients at Zhongnan Hospital of Wuhan University, and the normal tissues were from donors with accidental death. Tissues were obtained and stored following the protocol of Zhongnan Hospital Biobank. The study was conducted in accordance with the Declaration of Helsinki. Informed consent was obtained from all subjects and legally authorized representatives, and the approval of bladder tissues use was obtained from the Ethics Committee of Zhongnan Hospital (approval no. 2015029).

### Cell lines and culture

Human bladder immortalized epithelium cell line SV-HUC-1 (Cat. #TCHu169), BCa cell lines RT-4 (Cat. #TCHu226), T24 (Cat. #SCSP-536), UM-UC-3 (Cat. #TCHu217) and 5637 (Cat. #TCHu1) were got from Chinese Academy of Sciences, China. And BIU87 (Cat. #CL-0035) was obtained from the Procell Co., Ltd., China. RT4 was maintained in McCoy’s 5A medium (Gibco), UM-UC-3 was cultured in DMEM (Gibco), and all other cell lines were cultured in RPMI-1640 (Gibco). Fetal bovine serum (FBS, Gibco) was added to the culture medium to a final concentration of 10%.

### Transfection and plasmid construction

BCa cells were transfected with either *siRNA* or plasmid by Lipofectamine 2000 following the manufacture’s protocol. The sense sequences of *BORA*-*siRNA* were: *Si*-*1* (*siBORA/shBORA*), 5′-GGAGAUGUCAAGGAAUCAATT‐3ʹ; *Si*-*2*, 5′-CCAGUAAAUGCACUAACAUTT‐3ʹ; *Si*-*3*, 5′-GGAUAUGGUUGAUCCUAUATT‐3ʹ. The *si*-*control* (NC) was 5′-ACGUGACACGUUCGGAGAATT‐3ʹ. The BORA overexpression plasmid pECMV-3xFlag-BORA was obtained from GenePharma biotech company, China. To obtain stable *BORA* knockdown cell lines, UM-UC-3 and 5637 were infected with *lentiviral*-*control*-*shRNA* (*LV*-*NC*) and *lentiviral*-*BORA*-*shRNA* (*LV*-*BORA sh*), and then selected with 5 μg/ml puromycin (Sigma).

### RNA extraction and qRT-PCR

Total RNA was extracted from bladder tissues or cells using Qiagen RNeasy Mini Kit (Cat. #74101) following the manufacture’s protocol. The RNA quantity and quality were detected with NanoDrop^®^ ND-2000 UV–Vis (Thermo Scientific). Then 1 μg RNA was reverse transcribed to cDNA, which was then taken 1 μg to mix with primers, iQ™ SYBR^®^‑Green Supermix (Bio-Rad), and nuclease-free water to a final 20 μl volume to amplify by real-time polymerase chain reaction. The forward primer of *BORA* is 5′-GAGAAAAGCGATGCTGCTTGT‐3ʹ, and the reverse primer is 5′-GCTTCCGTTCCCATCTAAAAACA‐3ʹ. The forward primer of *GAPDH* is 5′-ACAACTTTGGTATCGTGGAAGG‐3ʹ, and the reverse primer is 5′-GCCATCACGCCACAGTTTC‐3ʹ.

### Cell proliferation and clonogenic formation assay

After transfection with *siRNA* or plasmid for 48 h, cells were transplanted into 96-well plates to proliferate for 1–5 days. As we described before, one plate was taken out every 24 h to treat with MTT and DMSO to detect the cell absorbance at 490 nm by microplate reader [22]. Clonogenic survival assay was performed with cells transfected after 48 h in 6-well plates. Specifically, 800 UM-UC-3 cells/well and 1000 or 3000 5637 cells/well were plated into 6-well plates to form colonies for 7–10 days. After that, colonies were fixed and stained to photograph and count for analysis.

### Flow cytometry analysis

After transfection for 48 h, cells were harvested in a 1.5 ml centrifuge tube and then washed once with cold PBS. Resuspending cells in 1 ml 1X DNA Staining solution and 10 μl Permeabilization solution (Multi sciences, Cat. #CCS012), vortexing for 5–10 s to mix well, and incubating 30 min at room temperature in a dark place. Then cells were tested on flow cytometry (Beckman, Cat. #FC500) on cell cycle at the lowest loading speed. For apoptosis analysis, as we described before, apoptosis detection kit (BD biosciences, Cat. #558547) was used according to its instruction [22].

### Transwell chamber assay

Cells transfected after 48 h were transplanted into the upper transwell chamber (Corning) with (4–6) × 10^4^ cells resuspended in 200 μl culture medium with no FBS. And the lower chamber added in 600 μl culture medium with 10% FBS to induce cell invasion and migration. Incubating for 24 h in cell incubator, cells in the upper chamber were removed. The go-through cells on the bottom side of the upper chamber were fixed and stained to photograph and count under the phase contrast microscope. The different step for invasion assay was that the transwell chamber was percolated with ECM Matrix gel (Sigma-Aldrich) before the cell seeding.

### Protein extraction and western blot (WB) analysis

RIPA with protease inhibitor and phosphatase inhibitor (Sigma-Aldrich) was used to lyse BCa cells. After 30 min on ice, cells were centrifuged at 4 °C and the supernatant was the protein solution. After a bath in boiling water for 10 min, the protein solution was ready to test. Protein was separated in SDS-PAGE gels, then transferred to PVDF membrane (Millipore), blocked with 5% milk, and incubated with primary (Additional file 1: Table S1) and secondary antibodies (Additional file 1: Table S2) sequentially. Bands were developed with chemiluminescence kit (Bio-Rad) and detected by Bio-Rad XRS^+^ Imaging system.

### Immunofluorescence (IF), immunohistochemistry (IHC), and hematoxylin and eosin (H&E) staining

Immunofluorescence staining for cells was described before [25]. For bladder tissues, immunofluorescence was performed by Biofavor Biotech, China. For IHC, paraffin section was sequentially going through deparaffinage, antigen retrieval, endogenous peroxidase inactivating, blocking with goat serum (Gibco), and incubating with primary (Additional file 1: Table S1) and secondary (Additional file 1: Table S2) antibodies. For H&E staining, the paraffin section of mice lung tissues and xenograft tumors were sequentially deparaffinized, rehydrated, stained with hematoxylin and eosin (Sigma-Aldrich), dehydrated and sealed. The slices were visualized and photographed with phase contrast microscope (Leica, Cat. #DMI 1).

### Xenograft and pulmonary metastasis mouse model

The 3-week-old male BALB/c-nu mice were purchased from Beijing HFK Bioscience Co., Ltd., China, and adapted 1 week to the animal facility of Zhongnan Hospital of Wuhan University. 4 × 10^6^ 5637 *LV*-*NC* or *LV*-*BORA sh* cells in 150 μl PBS were subcutaneously injected into the right flank to establish the xenograft model (n = 4). Xenograft tumor size was measured every 3 days (tumor volume = length × width^2^ × 0.5). And after 32 days of injection, tumor tissues were isolated from sacrificed mice to weigh and fixed in PFA for subsequent experiments. 1 × 10^6^ UM-UC-3 *LV*-*NC* or *LV*-*BORA sh* cells in 100 μl PBS were intravenously injected into tail vein to establish the pulmonary metastasis model (n = 3). Six weeks after injection, the fluorescence of pulmonary metastasis was monitored with FUSION FX7 Spectra Imaging system (Vilber), and the lung tissues were isolated to fix in 4% PFA for H&E staining. The animal experiment was in accordance with animal welfare and European animal care guidelines, and approved by the Institutional Animal Care and Use Committee at Center for Animal Experiment, Wuhan University (approval no. 2018152).

### Statistics

Representative data were from at least three independent iterations. Statistical analyses were carried out with SPSS 16.0. Two-tailed Student’s t-test and one-way analysis of variance were used to evaluate whether the difference of data was statistically significant, and p < 0.05 was considered significant.

## Results

### Upregulated BORA in BCa tissues

**Fig. 1:**
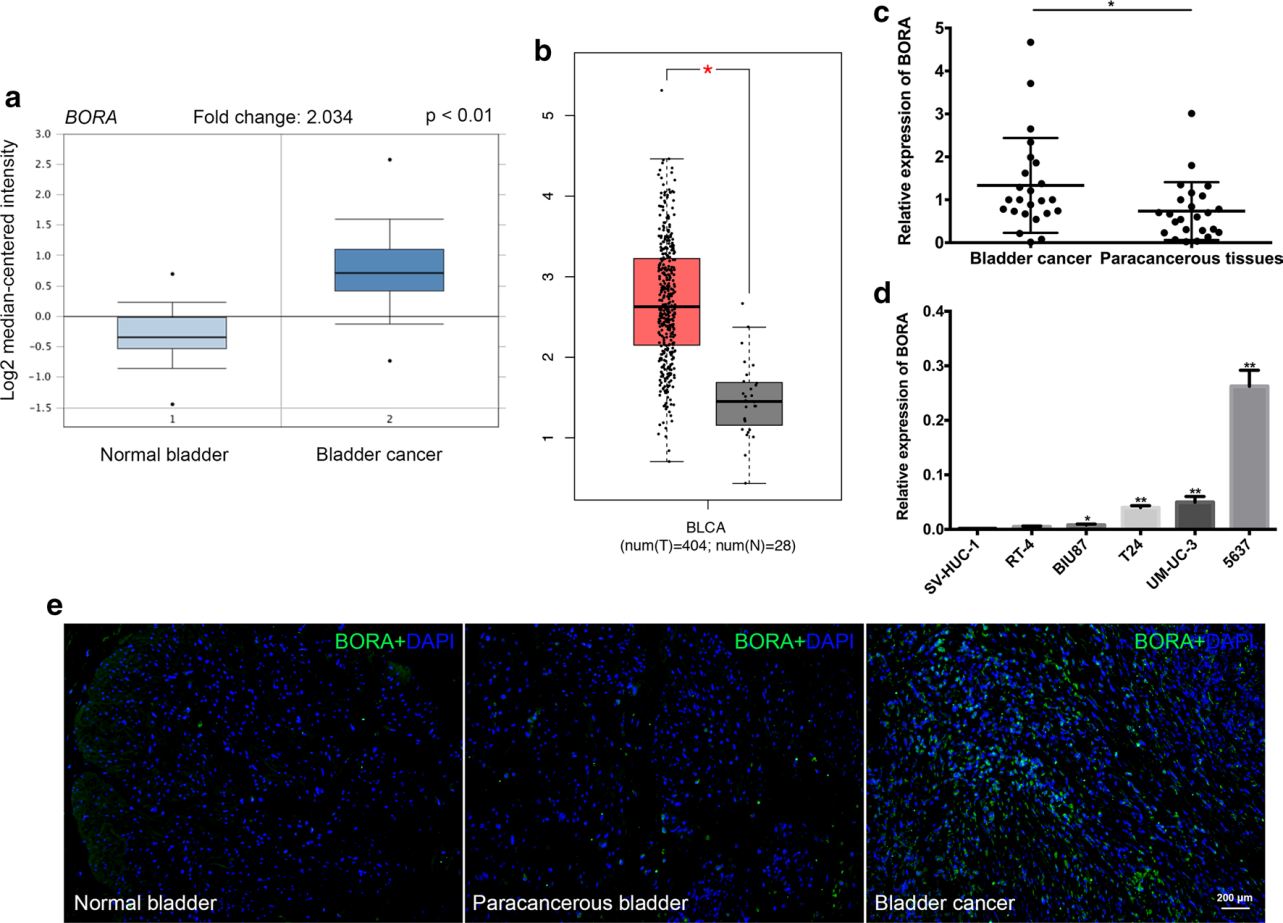
BORA was upregulated in BCa tissues. Upregulation of *BORA* was verified in Oncomine database (**a**) and GEPIA (**b**). **c** qRT-PCR results from our tissues indicated that *BORA* expression was significantly increased in BCa compared to matched paracancerous tissues. **d***BORA* expression in human immortalized normal bladder epithelium cell and BCa cell lines. *GAPDH* was the reference gene. *p < 0.05, **p < 0.01. **e** Representative IF staining of BORA (green) in normal bladder, paracancerous bladder and BCa tissues, with DAPI (blue) stained nuclei. The scale bar is indicated

We searched Oncomine database to find that mRNA expression of *BORA* was significantly increased in BCa compared to normal bladder (Fig. 1a). Gene Expression Profiling Interactive Analysis (GEPIA) is a website to easily achieve data visualization based on TCGA and GTEx projects [27]. The results showed that *BORA* was highly expressed in 404 tumor tissues compared to 28 normal tissues in bladder (Fig. 1b). To strengthen the results, we verified the mRNA and protein expression of BORA in BCa and paracancerous tissues collected from our hospital. qRT-PCR results revealed that *BORA* was upregulated in BCa tissues (n = 24) in comparison with paired paracancerous tissues (Fig. 1c). Furthermore, IF staining verified the upregulation of BORA in BCa tissues compared to paracancerous and normal tissues (Fig. 1e). All the results were consistent with our previous microarray results of 3 pairs of human BCa and normal bladder tissues [21]. We then detected the mRNA expression of *BORA* in BCa cell lines in comparison with human immortalized normal bladder epithelium cell line SV-HUC-1. Upregulated *BORA* was found in BCa cell lines, and among which, 5637 and UM-UC-3 were the most (Fig. 1d). Therefore, we chose 5637 and UM-UC-3 cell lines for the following experiments.

### Positive regulation of BORA in BCa cell proliferation

**Fig. 2:**
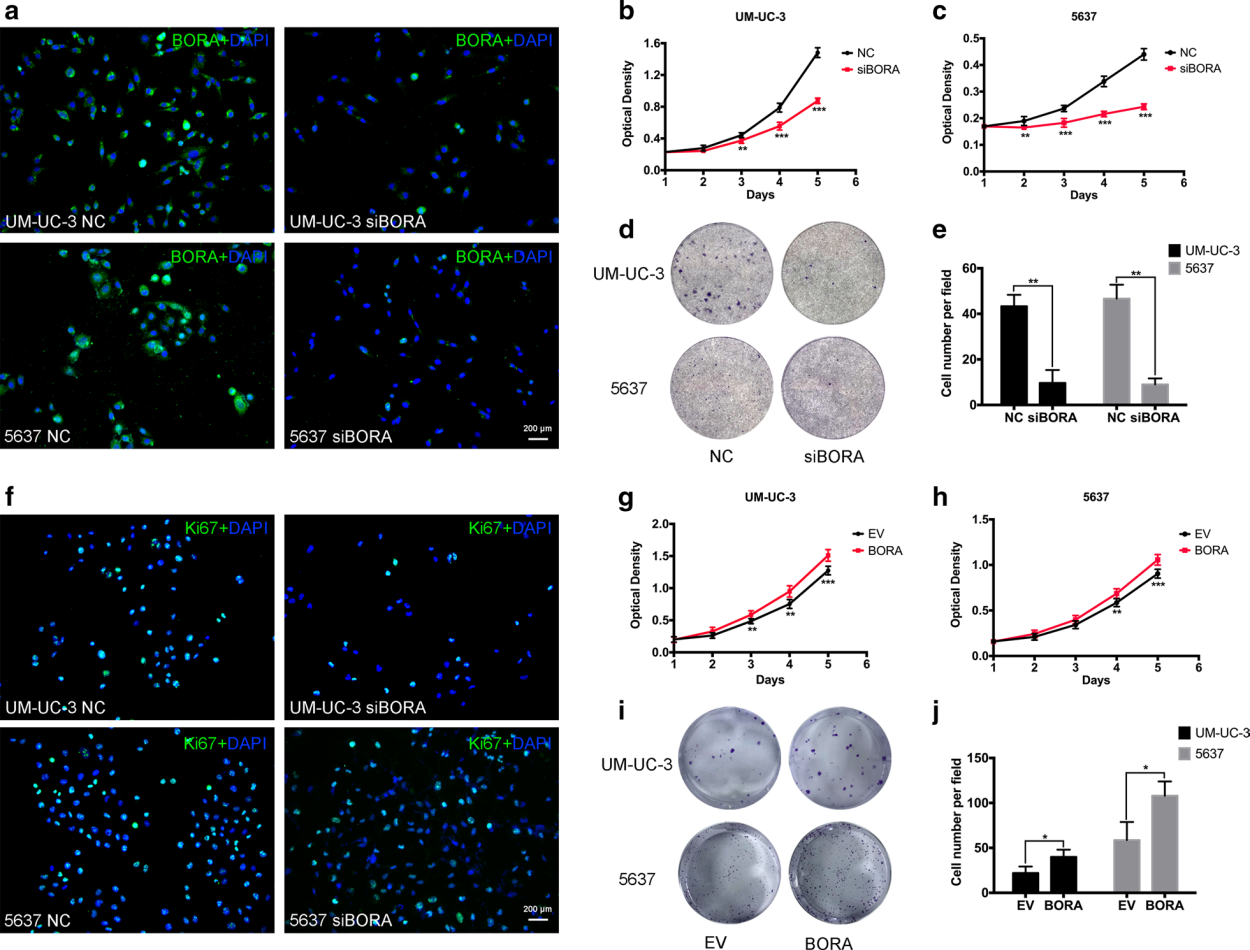
Influence of BORA on BCa cell proliferation. **a** IF staining confirmed the knockdown of BORA. The scale bar is indicated. **b** MTT assay to test the viability of UM-UC-3 and **c** 5637 cells transfected with *siBORA* or *NC*. **d** Influence of *BORA* knockdown on clonogenic formation efficiency and **e** statistical analysis of three independent experiments. **f** IF staining of Ki-67. The scale bar is indicated. **g** MTT assay to test the influence of overexpressed BORA on cell proliferation in BCa UM-UC-3 and **h** 5637 cells. And **i** the clonogenic formation assay (**j**) with statistical results. *p < 0.05, **p < 0.01, ***p < 0.001

To establish *BORA* knockdown cell model, UM-UC-3 and 5637 cells were transfected with three *BORA*-*target*-*siRNAs*. The knockdown efficiency was confirmed by qRT-PCR (Additional file 1: Figure S1A, B). We then chose the most efficient *Si*-*1* from the three *siRNAs* as the representative *siBORA* to knockdown *BORA* in BCa UM-UC-3 and 5637 cells, which was confirmed by WB (Additional file 1: Figure S1C) and IF assay (Fig. 2a). After knockdown of *BORA*, cell proliferation was significantly suppressed in UM-UC-3 (Fig. 2b) and 5637 (Fig. 2c) compared to the control NC group. Moreover, clonogenic formation results showed that *BORA* knockdown reduced the cell ability of colony formation (Fig. 2d), confirmed by statistical analysis (Fig. 2e). IF staining of the important proliferation marker Ki-67 [28], also revealed that *BORA* knockdown reduced Ki-67 positive cells (Fig. 2f). Furthermore, we constructed a BORA overexpression plasmid to figure out what was the effects of overexpressed BORA in BCa cells. Overexpression cell model was established by transfection of BORA plasmid into UM-UC-3 and 5637 cells, which was verified by qRT-PCR (Additional file 1: Figure S1D) and WB assays (Additional file 1: Figure S1E). Our results suggested that BORA overexpression had a proliferation-promoting effect in UM-UC-3 (Fig. 2g) and 5637 cells (Fig. 2h) compared to the control EV group and increased BCa cell clonogenic formation efficiency (Fig. 2i, j).

### Knockdown of *BORA* induced cell cycle arrest in G2/M phase

**Fig. 3:**
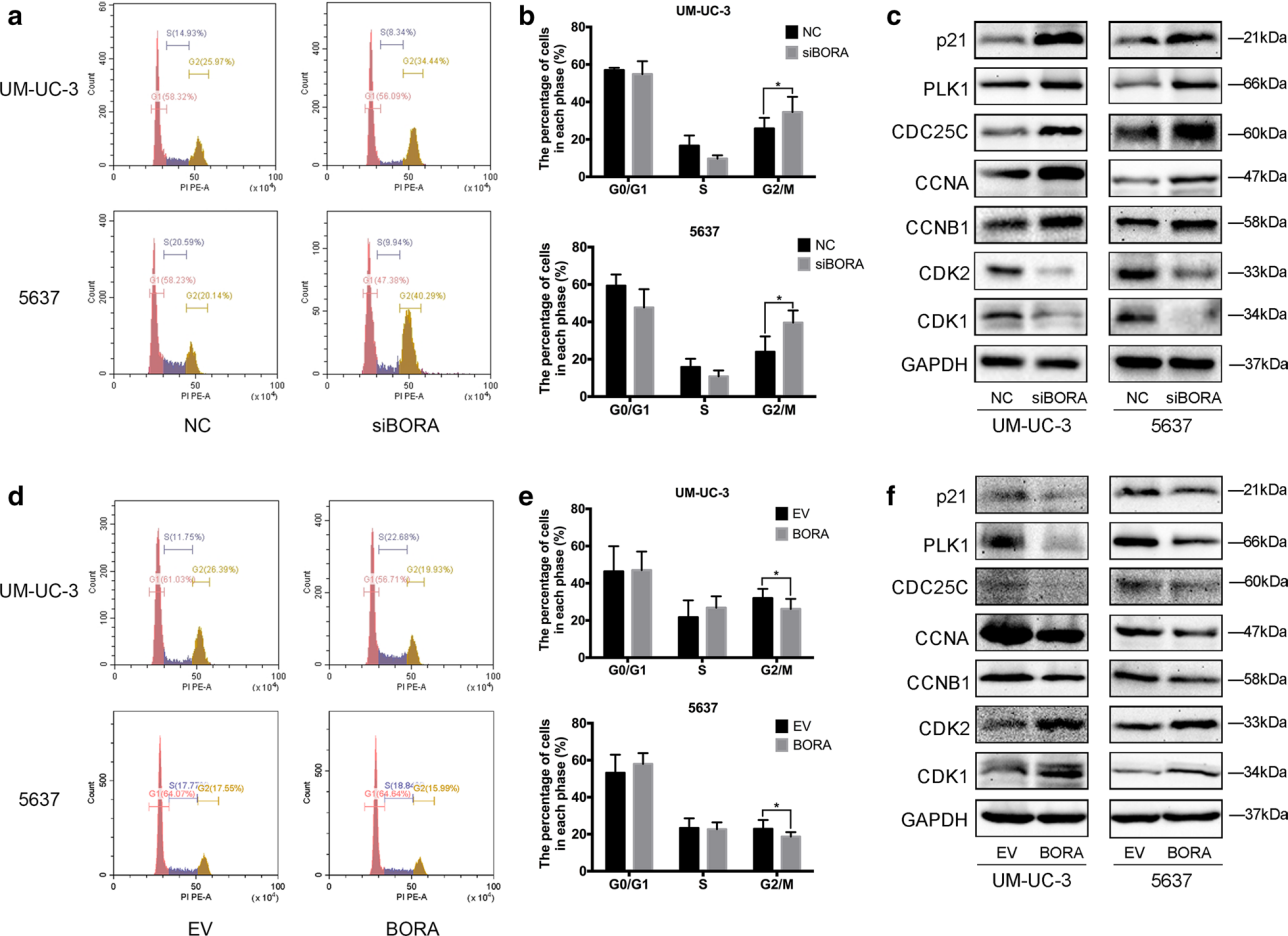
Cell cycle influence of BORA in BCa cells. **a** Flow cytometry cell cycle analysis of UM-UC-3 and 5637 cells with *siRNA* treatment. **b** Statistical analysis showed a significant increase in G2/M phase. **c** G2/M phase involved proteins and upstream regulators were analyzed by WB. **d** Cell cycle analysis after overexpression of BORA, **e** with statistical analysis. **f** WB assay of cell cycle related proteins. Values were shown with mean ± SD. *p < 0.05

Cancer proliferation is related to an active mitosis [29]. Then, flow cytometry was taken to assess the cell cycle conditions after transfection. Our results revealed that knockdown of *BORA* in UM-UC-3 and 5637 cells induced cell cycle arrest in G2/M phase (Fig. 3a), as indicated by statistical analysis (Fig. 3b). We also observed cell cycle related kinases CDK1 and CDK2 had a significant downregulation after *BORA* knockdown, while CCNA and CCNB1 protein had an upregulation as showed in Fig. 3c. PLK1/CDC25 was reported to have an important role in regulating the activation of CDK1 [30]. Also, p21 was a critical upstream regulator of cell cycle [31]. Their protein expression showed an obvious increase after *BORA* knockdown in UM-UC-3 and 5637 cells (Fig. 3c). We then detected the changes of apoptosis rate and found that BORA inhibition only was not enough to promote apoptosis in BCa UM-UC-3 and 5637 cells (Additional file 1: Figure S1F), which indicated a more complex regulation of apoptosis in cells. Furthermore, overexpression of BORA significantly reduced the proportion of G2/M phase in cell cycle (Fig. 3d), confirmed by statistical analysis (Fig. 3e). Also, the expression of cell cycle related protein CDK1 and CDK2 were upregulated, while CCNB1, CCNA, p21, PLK1, and CDC25C had a downregulation (Fig. 3f).

### BORA affected BCa cell motility with EMT-related proteins alteration

**Fig. 4:**
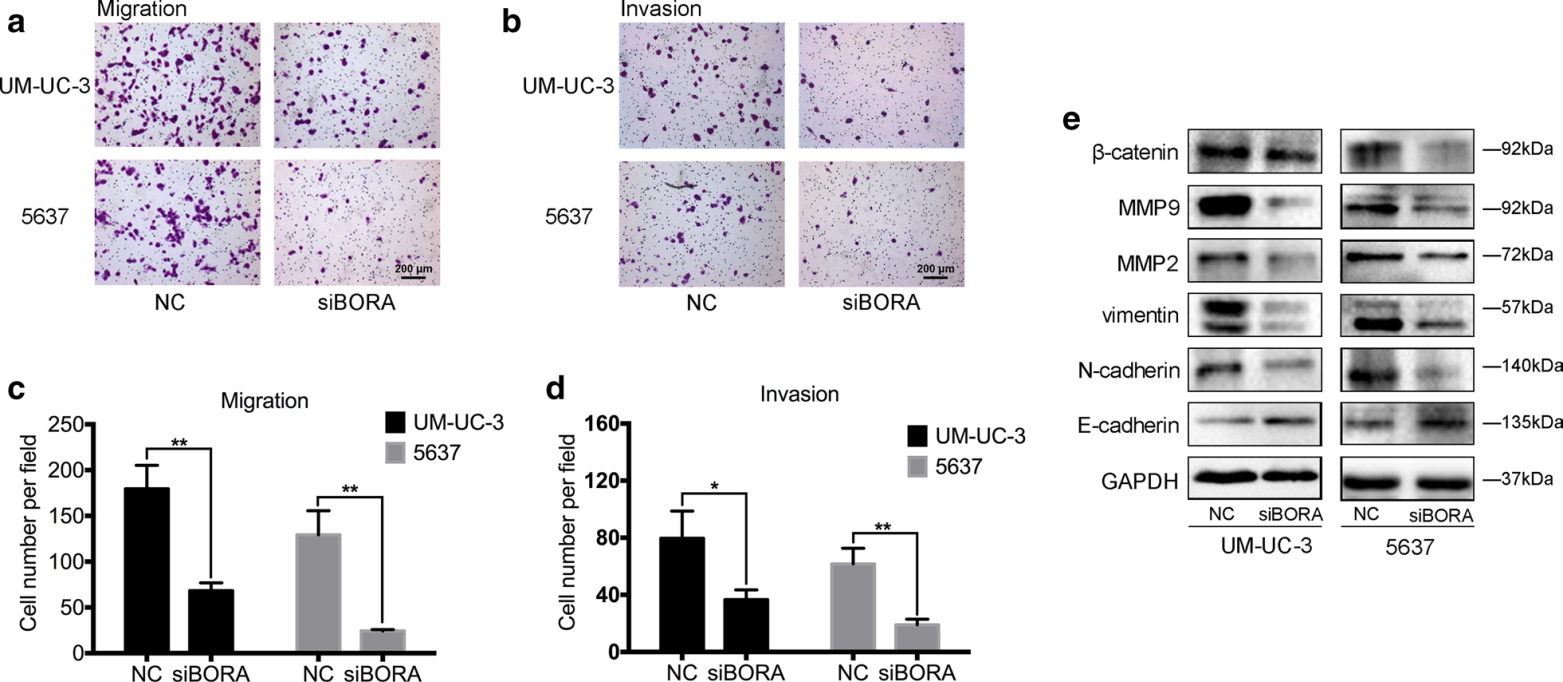
BORA regulated BCa cell migration and invasion via EMT. **a** Transwell migration and **b** invasion assays of UM-UC-3 and 5637 cells. **c** Statistical analysis showed a significant decrease of migration and **d** invasion rate of cells transfected with *siBORA*. **e** WB results showed the alteration of proteins related to EMT pathway. *p < 0.05, **p < 0.01

Interestingly, after knockdown of *BORA*, we noticed a reduction of transwell migration (Fig. 4a) and invasion rate (Fig. 4b) in UM-UC-3 and 5637 cells compared to NC group. Statistical analysis of the results from 3 independent experiments confirmed the significance of difference of migration (Fig. 4c) and invasion (Fig. 4d) rate reduction. The change of migration and invasion capacity in cancer usually associated with EMT process [32]. Therefore, we detected the EMT-related proteins and observed that the most important epithelial marker E-cadherin was upregulated, while mesenchymal and other EMT-related markers such as N-cadherin, Vimentin, MMP2, MMP9, and β-catenin were downregulated in *BORA* knockdown cells (Fig. 4e).

### *BORA* knockdown inhibited BCa cell proliferation and pulmonary metastasis in vivo

**Fig. 5:**
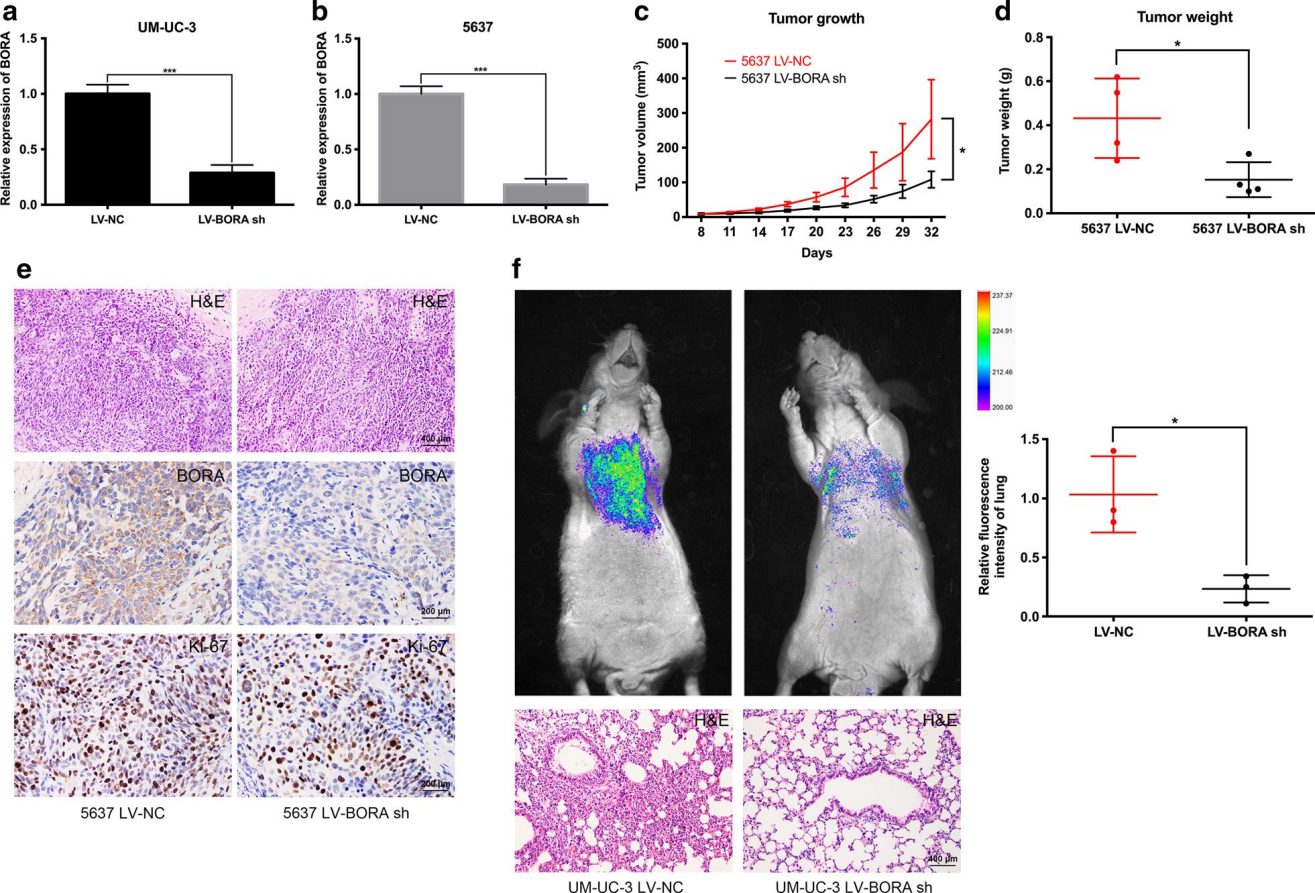
*BORA* knockdown inhibited BCa growth and migration in vivo. **a** Stable cell lines of *BORA* knockdown were established and confirmed by qPCR in UM-UC-3 and **b** 5637 cells. **c** Tumor volume was measured every 3 days. The x-axis represents days after transplantation. **d** The weight of the isolated tumor tissues. **e** Representative H&E and IHC staining of tumor tissues. The scale bar was indicated. **f** Fluorescence of pulmonary metastasis and statistics, and representative H&E staining of lung tissues. The scale bar was indicated. *p < 0.05

We conducted lentiviral packaging of *BORA shRNA* to establish stably downregulated BCa cells to verify the effect of BORA on cell growth and metastasis in vivo. The knockdown efficiency was confirmed by qRT-PCR in UM-UC-3 (Fig. 5a) and 5637 cells (Fig. 5b) before injection. In the xenograft mice transplanted with 5637, *LV*-*BORA sh* group showed a suppressed tumor growth compared to *LV*-*NC* group (Fig. 5c). Tumor tissues were isolated from mice after sacrificing to weigh, and the tumor weight of *LV*-*BORA sh* group was significantly lower than that of *LV*-*NC* group (Fig. 5d). Tumor tissues were then paraffin-embedded and stained with H&E (Fig. 5e). Moreover, IHC staining confirmed that BORA expression of the tumor in *LV*-*BORA sh* group was downregulated, and the proliferation marker Ki-67 also showed a decreased expression in *LV*-*BORA sh* group compared to *LV*-*NC* group (Fig. 5e). For pulmonary metastasis, *LV*-*BORA sh* UM-UC-3 and *LV*-*NC* UM-UC-3 cells were tail intravenously injected. Six weeks after injection, the fluorescence of pulmonary metastasis was detected, and the results suggested that knockdown of *BORA* significantly reduced BCa cell migration in vivo compared to *LV*-*NC* group (Fig. 5f).

## Discussion

As the activator of kinase Aurora A, BORA plays an important role in asymmetric protein localization, spindle assembly, and centrosome maturation during mitosis [8]. Most of the studies were focused on Aurora A and the interaction of BORA between Aurora A and PLK1 [20]. The function of BORA in cancer was seldomly researched especially in bladder cancer. Our previous microarray analysis of BCa and normal bladder tissues, which could be downloaded at Gene Expression Omnibus database with accession number GSE76211, showed upregulation of *BORA* expression in BCa tissues [21]. Therefore, in the present study, we firstly confirmed that the expression of BORA was upregulated in BCa tissues in comparison with normal bladder and paired paracancerous bladder tissues from our center combining with TCGA and Oncomine database (Fig. 1).

To explore the function of BORA in BCa, we established *BORA* knockdown and overexpression BCa cell model. Our results showed that knockdown of *BORA* could inhibit BCa cell proliferation and BORA overexpression could promote cell proliferation (Fig. 2). Cell growth is regulated by cell cycle activity, and BORA was reported to strengthen the activation of PLK1 to facilitate G2/M transition [33]. In our study, reduced BORA induced cell cycle arrest in G2/M phase, and upregulation of BORA decreased cells in G2/M (Fig. 3). G2/M entry is controlled by Cyclin-dependent kinase CDK1-CCNB and CDK1-CCNA. The activity of CDK1 is regulated by PLK1 through promoting CDC25 activation [30]. The activation of PLK1 relies on the phosphorylation of T210 residue at its T-loop [34], which is the result of Aurora A kinase effect with the cooperation of BORA [35]. However, some studies also showed that PLK1 and Aurora A conversely had an influence on BORA activity through posttranslational modifications [8, 36]. Recent studies demonstrated that CDK1 regulated the activation of BORA by phosphorylating 3 conserved sites located at the N-terminal part of BORA, which are Cyclin docking sites [20, 37]. Once mutated the three phosphorylation sites of BORA, PLK1 could not be activated by Aurora A at T210 on the T-loop. During DNA damage recovery, it is important for cells to maintain the G2 checkpoint, which provides cells with time for DNA repair or progress to apoptosis. Cairns et al. reported that BORA was significantly related to radiosensitivity by regulating DNA repair and MDC1, and BORA affected irradiation response via a different pathway from PLK1 [19]. We detected the expression of cell cycle proteins and found that PLK1 and CDC25C were upregulated, while CDK1 and CDK2 were downregulated after knockdown of *BORA*. Moreover, the protein changes were reversed after BORA overexpression in BCa cells (Fig. 3). GSK3β activation was reported to be important for BORA [38]. We noticed a downregulation of GSK3β after *BORA* knockdown in UM-UC-3 cells (data not shown). However, the exact mechanism of how BORA regulates and be regulated in the feedback loops still needs to be further explored.

Interestingly, after knockdown of *BORA*, we noticed that BCa cell migration and invasion were inhibited, which has not been reported anywhere else (Fig. 4). We observed upregulated epithelial marker E-cadherin in *BORA* knockdown cells and downregulated N-cadherin, Vimentin and other proteins associated with EMT pathway, which has been verified to play a critical role in migration and invasion of cancer cells [39]. But how reduced BORA exactly regulates EMT still needs further detections. To confirm our in vitro results, we established stable cell lines of *BORA* knockdown through lentiviral packaging. Xenografts and pulmonary metastasis mice model were established by subcutaneously and intravenously injecting BCa cells, respectively. In vivo, we also found reduced BCa cell growth and migration after knockdown of *BORA* (Fig. 5).

Recently, a large number of Aurora A and PLK1 inhibitors were reported to be evaluated in clinical trials as anticancer drugs but showed modest effect against solid tumors [40]. BORA, as the key intermediate protein of Aurora A and PLK1, was reported to be a potential biomarker for prognosis in lung, breast, and gastric adenocarcinomas [18]. Our results also showed that *BORA* knockdown could suppress BCa cell growth and migration both in vitro and in vivo. Researches about the effect of BORA in cancers are still too less. And how PLK1 and Aurora A inhibitors affect solid tumors after blockading BORA needs to be further investigated. As a signaling node of the BORA–PLK1–CDC25–CDK1 feedback loop and Aurora A-BORA-PLK1 axis, BORA is likely to have exciting prospects as a potential target.

## Conclusions

In conclusion, our study revealed that BORA positively associated with BCa cell growth and regulated cell cycle. For the first time, we found that *BORA* knockdown could suppress BCa cell migration and invasion possibly through EMT pathway. BORA has the potential to become a new biomarker and possible therapeutic target in BCa.

## Supplementary information

**Additional file 1: Figure S1.** Validation of *BORA* knockdown and overexpression. Knockdown efficiency of *BORA*-*siRNA* in **(A)** UM-UC-3 and **(B)** 5637 cells. **(C)** WB confirmed the knockdown of BORA. **(D)** BORA overexpression was verified by qPCR and **(E)** WB assay. **(F)** Apoptosis analysis of BORA knockdown. ** p < 0.01, *** p < 0.001, n.s. means no significance. **Table S1.** List of primary antibodies. **Table S2.** List of secondary antibodies.

## Data Availability

All data generated or analysed during this study are included in this published article.

## Abbreviations

BCa: Bladder cancer
EMT: Epithelial–mesenchymal transition
GEPIA: Gene Expression Profiling Interactive Analysis
H&E: Hematoxylin and eosin
IF: Immunofluorescence
IHC: Immunohistochemistry
PLK1: Pole-like kinase 1
WB: Western blot
