# Supplementary material for: BORA regulates cell proliferation and migration in bladder cancer
Source: Cancer Cell Int. 2020 Jul 6;20:290. doi: 10.1186/s12935-020-01392-8 (PMC7339433; doi:10.1186/s12935-020-01392-8)
Supplement: Supplementary file 1 — Additional file 1: Figure S1. Validation of BORA knockdown and overexpression. Knockdown efficiency of BORA-siRNA in (A) UM-UC-3 and (B) 5637 cells. (C) WB confirmed the knockdown of BORA. (D) BORA overexpression was verified by qPCR and (E) WB assay. (F) Apoptosis analysis of BORA knockdown. ** p < 0.01, *** p < 0.001, n.s. means no significance. Table S1. List of primary antibodies. Table S2. List of secondary antibodies. [file 12935_2020_1392_MOESM1_ESM.docx]

**Additional information**

**Additional figure**


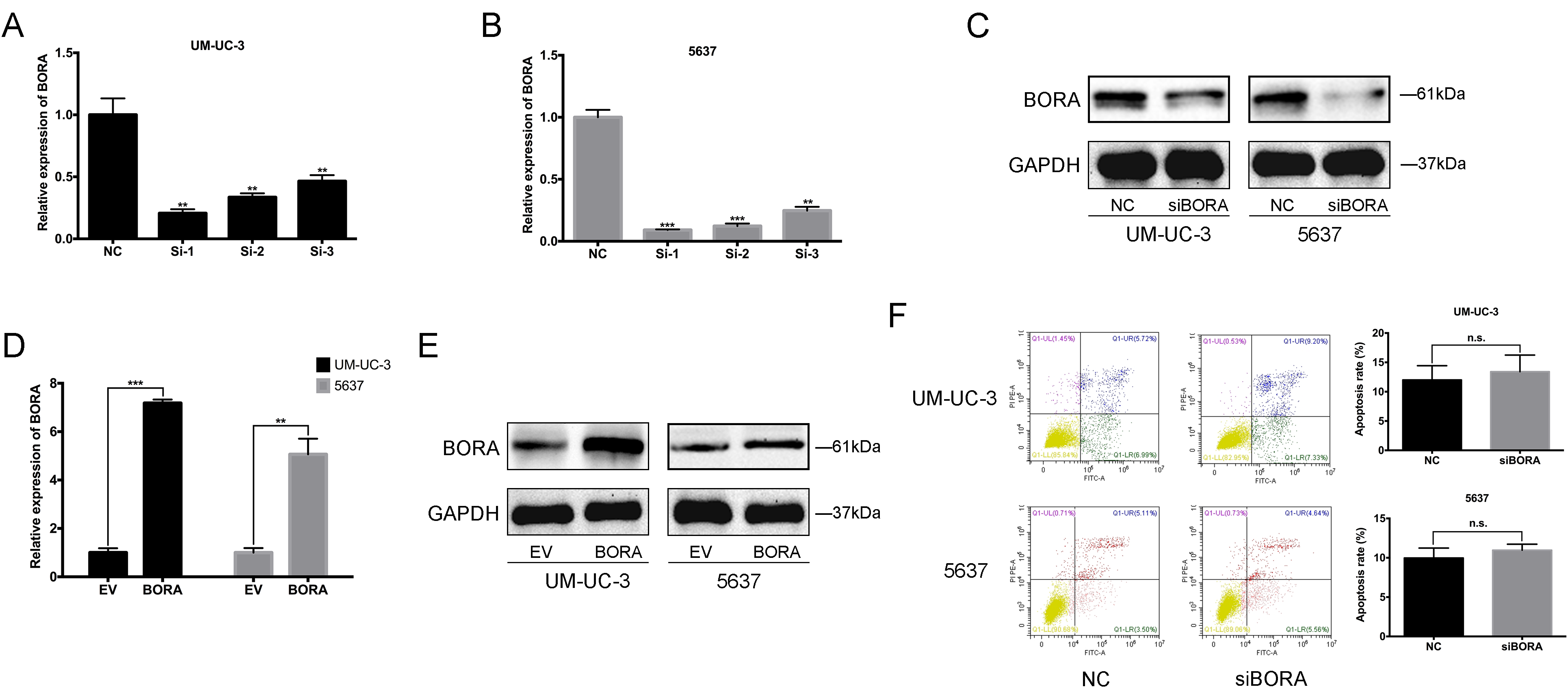


**Figure S1. Validation of *BORA* knockdown and overexpression.** Knockdown efficiency of *BORA-siRNA* in **(A)** UM-UC-3 and **(B)** 5637 cells. **(C)** WB confirmed the knockdown of BORA. **(D)** BORA overexpression was verified by qPCR and **(E)** WB assay. (F) Apoptosis analysis of BORA knockdown. ** p < 0.01, *** p < 0.001, n.s. means no significance.

**Additional tables**

| **Table S1. List of primary antibodies** | | | | |
| --- | --- | --- | --- | --- |
| Antigens | Species antibodies raised in | Dilution (IF or IHC) | Dilution (WB) | Supplier |
| BORA | Rabbit, monoclonal | 1:100 | 1:1000 | Abcam, ab182149 |
| GAPDH | Mouse, monoclonal | - | 1:2000 | Santa Cruz, sc-365062 |
| Ki-67 | Rabbit, monoclonal | 1:100 | - | Novus, NBP2-19012 |
| CDC25C | Rabbit, monoclonal | - | 1:1000 | Abcam, ab32444 |
| p21 | Rabbit, monoclonal | - | 1:1000 | Abcam, ab109520 |
| PLK1 | Mouse, monoclonal | - | 1:1000 | Abcam, ab17056 |
| CCNA | Rabbit, monoclonal | - | 1:2000 | Abcam, ab185619 |
| CCNB1 | Rabbit, monoclonal | - | 1:2000 | Abcam, ab32053 |
| CDK2 | Rabbit, monoclonal | - | 1:1000 | CST, 2546 |
| CDK1 | Mouse, monoclonal | - | 1:1000 | Abcam, ab18 |
| β-catenin | Rabbit, monoclonal | - | 1:1000 | CST, 8480 |
| MMP9 | Rabbit, monoclonal | - | 1:1000 | CST, 13667 |
| MMP-2 | Rabbit, monoclonal | - | 1:500 | CST, 13132 |
| Vimentin | Rabbit, monoclonal | - | 1:2000 | CST, 5741 |
| N-cadherin | Rabbit, monoclonal | - | 1:1000 | CST, 13116 |
| E-cadherin | Rabbit, monoclonal | - | 1:500 | CST, 3195 |

| **Table S2. List of secondary antibodies** | | | | |
| --- | --- | --- | --- | --- |
| Secondary detection system used | Host | Method | Dilution | Supplier |
| Anti-Mouse-IgG (H+L)-HRP | Goat | WB | 1:10,000 | Sungene Biotech, LK2003 |
| Anti-Rabbit-IgG (H+L)-HRP | Goat | WB/IHC | 1:10,000 | Sungene Biotech, LK2001 |
| Anti-rabbit IgG (H+L), F(ab)2 Fragment (AlexaFluor® 488 Conjugate) | Goat | IF | 1:100 | CST, 4412 |
